# COVID-19 Vaccine Motivation and Hesitancy among a Sample of African American, Afro-Caribbean, and African Respondents in the United States

**DOI:** 10.3390/vaccines12060571

**Published:** 2024-05-24

**Authors:** Shauna K. Elbers, Denise A. Vaughan, Yordanos M. Tiruneh

**Affiliations:** 1 School of Interdisciplinary Arts and Sciences, University of Washington, Bothell, WA 98011, USA; 2 School of Nursing and Health Studies, University of Washington, Bothell, WA 98011, USA; 3 Department of Preventive Medicine and Population Health, School of Medicine, University of Texas at Tyler, Tyler, TX 75708, USA; yordanos.tiruneh@uthct.edu; 4 Department of Internal Medicine, Division of Infectious Diseases, The University of Texas Southwestern Medical Center, Dallas, TX 75390, USA

**Keywords:** vaccination, Black, African, Caribbean, COVID

## Abstract

Understanding the motivations and decisions behind COVID-19 vaccine acceptance is crucial for designing targeted public health interventions to address vaccine hesitancy. We conducted a qualitative analysis to explore COVID-19 vaccine acceptance among diverse ethnic subgroups of Black Americans in the United States. This study investigates the 2021–2022 responses of 79 African American, Afro-Caribbean, and African respondents over the age of 18 in Washington State and Texas. Respondents were asked “Do you plan to get the COVID-19 vaccination?” Qualitative responses were analyzed by content category and ethnic subgroup. Of the 79 responses, 60 expressed favorable perceptions, 16 expressed unfavorable perceptions, and 3 expressed neutral perceptions. Dominant categories among participants in favor of the vaccine included personal health (26), concern for health of family/or community members (13), and desire to protect others (11). Among the 42 vaccinated African American respondents, the primary motivation was personal health (20). The 12 unvaccinated African American respondents cited fear of side effects as their dominate motivation. Caribbean respondents cited family or elders as motivation for their decision. African respondents were nearly unanimous in taking the vaccine (13/16), citing trust in health care, protecting friends and family, and personal health as reasons. Community and personal relationships were critical decision-making factors in accepting the COVID-19 vaccine, with African Americans having the strongest hesitancy.

## 1. Introduction

COVID-19 has disproportionately impacted Black communities in the United States, with the highest death rate (97.0 per 100,000) compared to all other racial groups, representing a nearly six-fold increase in the rate of death due to COVID-19 for African Americans [1,2]. However, the broad use of “African American” as a master status that homogenizes all Black experiences under one identity overshadows the ethnic distinctiveness within the Black experience [3]. Within health care, this lack of distinction poses a significant problem, as the terms “Black” and “African American” often mask ethnic and nativity subgroup differences in health outcomes [4,5], resulting in blanket treatment protocols and communications that may not be effective to address the needs of diverse ethnic subgroups. Given the evidence on the disproportionate impact of COVID-19 on Black communities and Violet Johnson’s [3] critique of the “monolithic” African American identity, this study examines the major influences guiding decisions regarding COVID-19 vaccine receipt in Black ethnic subgroups, including Afro-Caribbean, African American, and African respondents living in the United States. We explore the reasoning behind participants’ choices to either accept or decline the COVID-19 vaccine and assess if dominant patterns emerge within each ethnic subgroup. We also explore patterns to see if a dominant reasoning is identified by ethnic subgroup. We propose that by analyzing qualitative responses about why people do or do not take the COVID-19 vaccine, we will gain insights into the motivation for health care decisions communicated by respondents. We identified these motivations by examining the rhetoric employed in response to the two-part question “did you take the COVID-19 vaccine, why or why not”. By categorizing participant rhetoric into content categories, we identified patterns and themes underlying motivations shaping vaccination decisions.

In medical discourse, there is a longstanding body of literature on the rhetoric of medicine, which studies the use of language in the medical domain [6]. Most of the studies address the physician–patient dyadic relationship, mainly focusing on the effectiveness of communications [7] as well as examining how medical rhetoric influences decision-making in health-related matters [8,9]. While much of the research in the field of medical rhetoric focuses on the use of language to disseminate information, few studies have analyzed the message conveyed by patients or clients about their motivation for their health-related decisions [10]. The existing literature has failed to capture the perspectives of those individuals who opt not to seek health care, the role of the patient in describing the barriers to care, and the examination of differences among ethnic subgroups within racial categories. The current study aims to fill this gap.

Research indicates that medical mistrust is rooted in the historical maltreatment of African, Caribbean, and Black (ACB) individuals within the medical field [11] and is closely associated with personal experiences with discrimination [12]. This medical mistrust has been identified as a significant barrier to vaccine uptake in ACB communities including vaccines for HPV [13] and more specifically for COVID-19 [14]. Andrea Smith and colleagues [15] further expanded this discussion by showing that individuals who have experienced discrimination in health care had higher levels of medical mistrust overall, particularly regarding the COVID-19 vaccine, and are more likely to endorse COVID-19-specific conspiracy beliefs. While it has been well established that discrimination hinders Black communities from seeking health care, the ideological differences regarding the COVID-19 vaccine across Black subgroups are less explored. Public health practice requires persuasive communication skills to encourage individuals to seek treatment in the best interests of the general population [16]. Thus, understanding these ideological differences is critical to better communicate with ACB communities. Research on communication language concordance between patients and physicians supports the rationale for this study, suggesting that using just one approach in public health communication can alienate some subgroups [17,18]. Decisions about vaccines provide an opportunity to conduct discourse analysis on the motivation behind vaccine acceptance and refusal among ACB populations.

This study examines this rarely scrutinized discourse to identify the ethnic differences in discourse among Black respondents and evaluate the implications on public health communications. While research in persuasion often focuses on why a person pursues health care, it falls short of understanding the motivations behind refusing health care. Analysis in medical rhetoric is primarily focused on patients and clients rather than those who do not receive health care [19]. This gap in the literature is amplified when considering Black populations. Therefore, this study illuminates the rationale behind vaccine hesitancy and highlights the ethnic subgroup differences in motivation.

## 2. Methods

### 2.1. Data Source

Data for this study came from the RHEALTH study, a multi-year survey conducted between 2018 and 2022, with both qualitative and quantitative questions (see Tiruneh, Anwoju, Harrison, Garcia, and Elbers, 2024 for a brief description of the study) [20]. This analysis was conducted on the COVID-19 questions collected between 2021 and 2022. The survey specifically focuses on respondents who identify as Black and who answered the COVID-19-related items with responses sufficiently detailed for content analysis (n = 16 African, n = 6 Afro-Caribbean, and n = 50 African American). This study was submitted to our institutional review board and received an exemption status, indicating low or minimal risk to respondents. A consent statement was still presented at the beginning of the study with instructions indicating that completion of the survey implied consenting to participate in the study. Two questions are the focus of this study. The first question “Do you plan to get the COVID-19 vaccine?” originally had five categorical choices which were later recoded into a binary variable by grouping respondents who answered either “I have already received the vaccine” or “I plan to get the COVID-19 vaccine” as yes. The 3 remaining responses; “I will not get the COVID-19 vaccine”; “I don’t know if I will get the COVID-19 vaccine, I am still thinking about it”; and “I don’t know if I will get the COVID-19 vaccine, I need more information” were coded as no. The second question was open-ended to allow participants to express multiple motivations influencing their decision-making regarding the COVID-19 vaccine, allowing for short answers or explanations. The participants were asked to share the reasons why they chose to receive the vaccine, decline the vaccine, or remain undecided. From the total of n = 239 respondents, 112 answered the COVID-19 questions and 79 provided a response to the second question beyond yes or no. Of the 79 responses we examined, 72 responses provided enough material to indicate a content category.

### 2.2. Data Analysis

We employed grounded theory and content analysis to analyze the qualitative data. Grounded theory is used to examine underlying patterns in data and generate theoretical explanations or prepositions regarding the reasons for the observed patterns [21]. Content analysis was simultaneously employed to identify themes within the responses and to highlight the rhetoric that was identified as important by the respondent. We then organized the responses into content categories. Both the rhetoric participants used and the identified content categories illuminated the motivations of the respondents. Finally, we identified key motivations for respondents and further grouped them into ethnic groups to test whether motivations were consistent across ethnic subgroups.

## 3. Results

### 3.1. Content Categories Identified

The results of the content analysis revealed a remarkable finding: the categories important in persuading individuals to take the vaccine were remarkably similar to those persuading respondents to reject it (Table 1). In this study, whether the subject accepted or rejected the vaccine was not the key variable. Instead, the underlying motivations were the key indicator of how the subject was persuaded. The most frequently cited category was personal health. Here, respondents valued taking or refusing the vaccine motivated by protecting their personal health. The respondents said that they wanted to be healthy, so they took the vaccine, or they feared for their health, so they refused it. The second most common category was the specific other. Here, respondents indicated their parents or close family members, and the responses were more intense than in the personal health category where the respondents were more circumscribed. For example, respondents articulated their motivations through statements such as: “I am a wife and mother, and it’s absolutely critical that I protect my family. I also work in a contact center with hundreds of people”. Such statements illustrate a sense of personal responsibility to safeguard loved ones. Another respondent stated, “I have been vaccinated against COVID-19 and I am responsible for my family and my safety”, indicating personal and specific responsibility. Another participant expressed their motivation for familial or communal protection as, “I wanted to protect myself, family, and community from the possibility of contracting the virus and further spreading it”. This saying demonstrates a broad sense of responsibility to the community, highlighting the interconnectedness of personal health with public well-being. The third most frequent category pertained to abstract concepts, such as a reference to humanity. Additionally, belief in science emerged as a specific and common category and a few respondents sought a return to normalcy.

### 3.2. Personal Health

Protection of individual or personal health was the single highest response category in both those who chose to vaccinate and those who chose not to vaccinate. Of the 62 people who chose to vaccinate, 26 predominantly identified protection of their individual health. The self-health group responses included “Vaccinations will make me healthier”. Others included a connection to the specifics of how a vaccine can protect them: e.g., “…because it reduces the chances of me getting worse symptoms as opposed to the symptoms I can get now that I have the antibodies”. Here, the respondents are motivated by fear, a pathos response. Personal health was also a persuasive factor for 15 respondents who indicated that they chose not to vaccinate. Responses for the unvaccinated group were significantly shorter than those for the vaccinated group. A couple of individuals responded extremely simply with “I don’t want to” or “no” and “Vaccine hesitancy”.

### 3.3. Specified Other

The second largest theme that emerged from respondents who chose to be vaccinated was coordination between health-specified family or community members (13 responses). This connection to the community appears to be a major motivation for respondents to take the COVID-19 vaccine. Responses encompassed desires to protect specific other individuals, including family and community elders. Responses also included statements of personal responsibility and moral obligation: “To protect my elders and myself”. A few anti-vaccine respondents indicated specific distrust. Responses included specific reference to negative medical experiences in the ingroup identity group: e.g., “Tuskegee experiments and studies that correlate vaccination with autism create distrust. Also too early to fully understand future complications of the COVID-19 vaccine”. Here, the respondents are influenced by logical reasoning coupled with an appeal to emotion. The respondents draw on historical examples to apply to themselves and are motivated by the fear that history inspires.

### 3.4. Nonspecific Other

Respondents who indicated the nonspecific other include comments such as “I chose to get the vaccine because I believe it is the right thing to do. I would hate to get my family sick, so I’d rather protect myself and my family” and “the greater good. I wanted to do my part in keeping our people healthy. Specifically for my Black people, who already die disproportionately in a myriad of ways; some unique ways”. These responses that include community connections indicate the value of the community in decision-making.

### 3.5. Health of a Specific Other

Health of a specific other was another key content area. This set of responses indicated the importance of individual responsibility and family or community connection. Specific responses included personal responsibility, which is connected to the care of specific others: e.g., “I am a wife and mother, and it’s absolutely critical that I protect my family”.

### 3.6. Belief in Science

The final category indicated a belief in science and health care professionals. Responses ranged from specific details about the transmissibility of the disease to the role of identity as a member of the health care community. Responses here included the following: “I am a scientist, and I believe getting the vaccine will get us out of the pandemic sooner”. Others included specifics about how the disease works. “In order to prevent further contamination, we must take preventative measures that decrease our chances of carrying the virus” and “I chose to get the vaccine due to researching more information about it. Also I waited a few months after other people got the vaccine to try to see if there were any severe or worrying symptoms”. Here, faith is in the medical establishment. This is reminiscent of Daniel Freeman et al.’s study [22] where they argue that good experiences in health care are a strong determinant of willingness to be vaccinated. References to the ingroup are also present. One respondent said, “While it has been developed pretty quickly, Pfizer hasn’t had any real bad reports come out of its testing. I am also a supporter of medical science and am encouraged that a Black woman did help in the development of some of these vaccines”. This is a direct reference to a researcher as a member of the ingroup. Since one of the COVID researchers was a Black woman, the validity of the vaccine was increased in the mind of the respondent, which increased the willingness to take the vaccine.

### 3.7. Other

Several respondents indicated a desire to return to normalcy or cited other barriers to getting the vaccine. Those who wanted a return to normalcy responded with comments like “Not enjoying life yet”. Those who were hesitant indicated “vaccine hesitancy” or “still thinking” without referencing their reasoning. Two cited specific barriers: “The number of vaccines is limited” and “Don’t have the time”. A final group simply responded that they did not take the vaccine.

### 3.8. Word Count Analysis

Researchers also conducted a Word Count Analysis on the COVID-19 responses (Table 2). The largest category is respondents who used first-person pronouns such as “I” or “me”, which occurred 42 times out of 72 responses. This indicates a strong personal connection and involvement in the answer. The responses also suggest active engagement in their decision-making. The motivations are personal. The word “vaccine” appeared 23 times. This does indicate that the respondents were directly focusing on the vaccine itself and not health care in a broader sense. The term “health” occurred 16 times. This is often a connection to personal or family health. “Family” and “community” are also frequent terms, which are clearly tied to the content category of the specific other. The repetition between word frequency and content category emboldens the strength of this category. The final word count category, “want”, is particularly important as it ties to our connection with means of persuasion. Twelve times, the respondents used the term “want” such as “I want a return to normalcy”. This indicates that the respondent is looking toward a change in the future. In persuasion, if the goal is to change action, the audience must first see a potential better future and then be willing to work to get there. At least 12 times, these respondents embodied that desire for change.

### 3.9. Ethnic Subgroup Differences

Differences also emerged among African American, Afro-Caribbean, and African respondents (see Table 3). We found that for African Americans, there were 42 affirmative and 10 no responses where the predominant theme among those who responded affirmatively was to protect their personal health (20). The second most common response pertained to health care with distinct perspectives on trust in medicine and concerns about side effects (12). Respondents who expressed trust in medicine cited reasons like, “I work in public health and really want to get to herd immunity also want to make sure I don’t spread disease in my community/family” and “I worked in pharmaceutical industry and trust that vaccines work”. Conversely, those who expressed distrust in the vaccine mentioned concerns about potential side effects. Comments ranged from “I’m worried about the side effects of the vaccine” to a call for others not to get vaccinated, “Do not vaccinate against COVID-19, because the vaccine has side effects”.

Caribbean American respondents were evenly split: three took the vaccine and three did not. For the yes themes, two cited family (parents’ demands and to protect elders). For the no themes, two also cited health care (Tuskegee and vaccines cause autism, want to watch for results, and vaccines increase the risk of contracting COVID-19). This group was encouraged to take the vaccine by their family/community, but the respondents who chose not to be vaccinated were influenced by health care. Black African respondents dominantly chose to take the vaccine. Thirteen respondents took the vaccine and three did not. The top theme for Black African respondents was family and community. The secondary theme was the protection of oneself.

## 4. Discussion

Indicators of participant motivations regarding acceptance or refusal of the COVID-19 vaccine can be found in both the rhetoric respondents used and in the categories of responses identified. This underscores the necessity for public health professionals to identify specific factors motivating each subgroup to effectively influence behavior. Recognizing that identical categories of motivation can either encourage or hinder vaccine uptake, contingent upon motivation differences by subgroup, is critical in crafting targeted public health messaging. Furthermore, while there are similar characteristics and motivating categories across participants, ethnic subgroup differences in primary motivation are evident. This means that a respondent’s ethnic subgroup determines which value is a primary factor in deciding whether or not to take the vaccine. In terms of ethnic subgroups, African Americans’ top motivating content category was personal health followed by family. For Caribbean Americans, family was the top motivating content category to either take or reject the vaccine. For Black Africans, family was the top content category, followed by self-health.

This illustrates the high degree of complexity involved in determining whether to take the vaccine or not beyond the standard public health racial groupings and without considering the impact of individualism vs. collectivism cultures [23]. This further means that public health messaging, intervention, and practice may fail to adequately address Black communities by assuming that primary motivating factors for vaccine compliance and refusal are similar across ethnic subgroups. This is consistent with Celia Roberts and Srikant Sarangi’s finding that communication can be aided by tracking how the social construction of meaning influences the acceptance of the persuasive message [8].

Given that COVID-19 spreads via close contact, tailoring community-specific messaging is integral to fostering the widespread acceptance of vaccine interventions [24]. The current study has significant implications for crafting targeted public health messages targeted at categories that are proven to be most persuasive for community groups. The importance of our findings can be best visualized by examining the intersecting processes outlined in Alan Monroe’s Motivated Sequence [25]—a Communications Theory and the Theory of Reasoned Action, a Health Behavior Theory. These categories confirm the efficacy of leveraging culturally specific analytic themes to shape public health messaging [14,26]. However, our study highlights the need to move beyond monolithic racial categories and embrace a more nuanced understanding of cultural diversity within communities.

**Figure 1:**
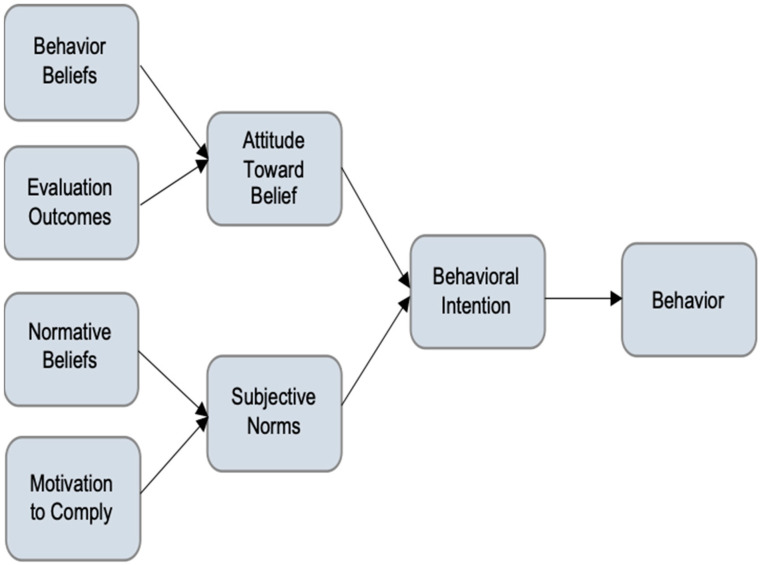
Theory of Reasoned Action.

There are five steps in Alan Monroe’s Motivated Sequence: attention, need, satisfaction, visualization, and a call to action [25]. The first step is to gain the audience’s attention (Why should I listen/trust you?). The second is to identify the problem that is of concern to the audience (Should something be done?). Satisfaction is to fulfill the need with a step-by-step solution (What should I do?). Visualization allows the audience to visualize a better future after the solution is applied (I can see this benefiting the people I care about). The fifth step, call to action, is what the audience should do to obtain a better future (I will engage in the behavior). The Theory of Reasoned Action suggests that a person’s health behavior is determined by their intention to perform a behavior (see Figure 1) [27].

A person’s intention to perform a behavior (behavioral intention) is predicted by (1) a person’s attitude toward the behavior and (2) subjective norms regarding the behavior. Subjective norms are the result of social and environmental surroundings and a person’s perceived control over the behavior. Generally, a positive attitude and positive subjective norms result in greater perceived control and increase the likelihood of intentions governing changes in behavior [27]. Here, our content analysis results can help us to identify what the subjective norms of the subgroup will be regarding specific messages. More specifically, the word count content analysis tells us what rhetoric the respondents use and how individually connected each respondent is to the message.

**Figure 2:**
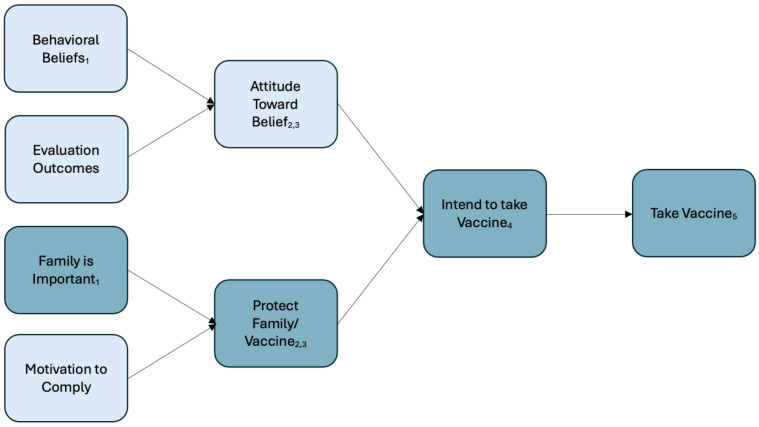
Content-Specific Theory of Reasoned Action with reference to Monroe’s Motivated Sequence. Note: Monroe’s motivating sequence in the subscript: (1) “Why should I listen to you?”, (2) “Should something be done?”, (3) “What should I do?”, (4) “I can see this benefiting the people I care about”, and (5) “I will engage in the behavior”.

A comparison between the Theory of Reasoned Action (Figure 1) and the content-specific Theory of Reasoned Action (Figure 2) illustrates the practical application of these findings. Figure 2 demonstrates that for African and Afro-Caribbeans, the content-specific findings revealed that the normative belief “family is important” led to the subjective norm to protect family which then leads to the behavioral change of adhering to vaccine uptake. In other words, our analysis reveals that the persuasive message for African and Afro-Caribbean respondents centers around the normative belief demonstrated by “family is important”. The implication for public health is profound; the persuasive locus lies within the normative belief regarding the importance of family. This could also manifest as a decision not to take the vaccine; however, the pathway is still the same in that the normative belief is “family is important”.

Traditional appeals to logos in messaging are less likely to be effective. Respondents indicate a strong personal attachment to family or community. Pathos appeals activate those personal relationships, which for the respondents are so important to their decision-making process about receiving the COVID-19 vaccine. For instance, our research identifies family as the most influential motivating factor for Africans and Afro-Caribbeans. Therefore, a public health message that emphasizes the importance of families is likely to resonate deeply with these demographic groups.

Finally, the surprising finding in our study is the unexpected similarities between motivations among respondents, regardless of whether they accept or refuse vaccination. These similarities suggest that the underlying motivation itself holds greater importance than the result of the motivation. Extending this notion to ethnic subgroups, although motivations were different between ethnic subgroups, the core motivations—such as the desire to protect family or prioritize self-health—were consistent regardless of whether the respondent accepted or refused the vaccine. Regardless of the motivator, there are clear subgroup differences that need to be considered in public health messages.

Discourse analysis examines how people construct meaning in interpersonal interactions, moment to moment. Similarly, concordance in a medical setting allows patients and medical care providers to share communication assumptions. In the same way, public health professionals must identify which categories resonate persuasively with a specific audience and employ effective pathos appeals to persuade them. The importance of these findings can be underscored by Irene Clark and Ronald Fischbach as cited by Jennifer Malkowski and Lisa Meloncon who assert that “the effectiveness of (our) work” [28] (pp. 20–21) as public health professionals rests in our ability to persuade our audience that our message is relevant and useful to them when making personal decisions about taking action to change.

The need for qualitative research to understand the cognitive determinants behind vaccine hesitancy has been well documented. Psychological reasons behind vaccine hesitancy include confidence and complacency, two common factors that are connected to people’s fears and concerns regarding vaccination [29]. More specifically, as Razai et al. argue, both ethnic and socioeconomic status aggravate the factors leading to vaccine hesitancy, and trust-building through consistent non-stigmatizing messages can help overcome historical mistrust among Black populations [30]. Targeted vaccine information/communication is key to effectively responding to concerns regarding the vaccine, thus alleviating hesitancy [31]. This study identifies the critical motivation differences among Black ethnic subgroups that can inform public health messages to build trust.

This research is subject to a few challenges including the brevity of responses. Longer responses often reveal more details and therefore more information about the respondent’s approach. There were also a limited number of respondents, which constrains the generalizability of the results. Repetition of this work could substantiate the research findings. Further research is required to understand how knowledge of primary motivating factors might alter public health communications to Black subgroups, enhancing receptivity to health care practitioner’s messages. Further research is also needed to understand the role of provider congruence [32] as a potential facilitator of effective messaging to Black ethnic subgroups.

## 5. Conclusions: Public Health Implications

When terms such as “Black” and “African American” are used in health research, it masks ethnic and nativity subgroup differences in health behavior motivation. As health professionals, we must understand the needs of our population how to communicate with them and the rhetorical method that is important to them.

## Figures and Tables

**Table 1:** Grouping of initial codes to form themes.

Theme	N of Participants	N of Transcript Excerpts	Sample Quote
Personal health: Those who cited personal health were fairly non-specific. They wanted to be healthier.	72	44	“I feel as though getting it is better than not getting it, because it reduces the chances of me getting worse symptoms as opposed to the symptoms I can get now that I have the antibodies.”
Specific Other: This set of responses indicated the importance of individual responsibility and the family or community connection.	72	8	“To protect my elders and myself” “I wanted to do my part in keeping our people healthy. Specifically for my Black people, who already die disproportionately in a myriad of ways; some unique ways.” “Tuskegee experiments and studies that correlate vaccination with autism create distrust. Also too early to fully understand future complications of the COVID-19 vaccine.”
Health for unspecified other: Eleven respondents indicated they took the vaccine because of a desire to protect others without specifying the others they were protecting.	72	11	“I currently work in healthcare and I feel as if it’s my responsibility as a human to do my part in wiping this plague from the Earth.”
Belief in science or health care	72	7	“I am a scientist and I believe getting the vaccine will get us out of the pandemic sooner.” ”I am also a supporter of medical science and am encouraged that a Black woman did help in the development of some of these vaccines.”
Desire to return to normalcy	72	2	“I want to travel again.” “I really wanted life to go back to normal.”
Nonspecific No	72	2	“Vaccine hesitancy” and “Still thinking.”
Other barriers	72	3	“The number of vaccines is limited.” “Don’t have the time.”

**Table 2:** Word count analysis.

”I” or “Me”	42	Community	3
Vaccine	23	Prevention	4
Health/healthy	16	Trust (1 trust; 2 do not trust)	3
Family	9	Want	12

**Table 3:** Ethnic subgroup vaccination status.

	Not Vaccinated	Vaccinated or Intend to
African American	10	42
Afro-Caribbean	3	3
African	4	17
Total respondents	17	62

## Data Availability

The original contributions presented in the study are included in the article, further inquiries can be directed to the corresponding author.
